# Digital support systems to improve child development in Peru: A cluster-randomized controlled open-label trial

**DOI:** 10.1126/sciadv.aeb9403

**Published:** 2026-03-04

**Authors:** Günther Fink, Dana Charles McCoy, Lena Jäggi, Kristen Hinckley, Andreana Castellanos, Maria Luisa Huaylinos Bustamante, Leonel Aguilar, Maria Catalina Gastiaburu Cabello, Milagros Alvarado, Sarah Farnsworth Hatch, Marta Dormal, Jorge Cuartas, Ce Zhang, Stella Hartinger Peña, Daniel Mäusezahl

**Affiliations:** ^1^ Swiss Tropical and Public Health Institute, Allschwil, Switzerland.; ^2^ University of Basel, Basel, Switzerland.; ^3^ Harvard Graduate School of Education, Cambridge, MA, USA.; ^4^ Universidad Peruana Cayetano Heredia, Lima, Peru.; ^5^ Afinidata, Fort Collins, CO, USA.; ^6^ Institute for Computing Platforms, Federal Institute of Technology Zurich, Zürich, Switzerland.; ^7^ Department of Applied Psychology, New York University, New York, NY, USA.; ^8^ Centro de Estudios Sobre Seguridad y Drogas (CESED), Universidad de los Andes, Bogotá, Colombia.; ^9^ Department of Computer Science, University of Chicago, Chicago, IL, USA.

## Abstract

Digital technologies have the potential to transform early childhood development (ECD) interventions by delivering personalized support at scale. We conducted a cluster-randomized controlled trial in rural Peru to evaluate the effectiveness and cost-effectiveness of an artificial intelligence–supported digital parenting chatbot as well as traditional home visits as interventions to improve child development. Among 2461 caregiver-child dyads, both the digital and home-visiting interventions improved child development outcomes at 2.5 years of age, with standardized effect sizes of 0.11 and 0.17, respectively. At ^1^/_15_ of the cost of in-person support, the digital intervention yielded superior cost-effectiveness. These findings suggest that digital platforms can be a viable, scalable alternative to support children’s development in resource-constrained settings.

## Teaser

An AI parenting chatbot boosted early childhood development in rural Peru at far lower cost than traditional home visits.

## INTRODUCTION

Globally, more than 250 million children under the age of five years are at risk of not reaching their developmental potential because of poverty, limited stimulation, and inadequate early caregiving (*1*). A growing body of research highlights the importance of early life investments for long-term health, education, and economic outcomes (*2*, *3*). While home-visiting programs have demonstrated substantial benefits in early childhood development (*4*), they remain resource-intensive and difficult to scale in low- and middle-income countries (*5*–*7*).

In recent years, artificial intelligence (AI) has enabled a previously unavailable generation of digital tools that can deliver timely, personalized, and developmentally informed guidance to caregivers at low cost (*8*). These innovations represent a potential breakthrough in efforts to support young children at scale, particularly in underserved regions. However, the effectiveness of AI-driven digital parenting tools has not been rigorously evaluated in low- and middle-income country contexts.

We report results from a large-scale randomized controlled trial in rural Peru assessing the impact of a digital parenting chatbot on early child development outcomes. We compare the impact of this intervention to a traditional home-visiting program following national guidelines. This study addresses two key questions: Can AI-powered digital support improve child development? How does its effectiveness compare with in-person programs?

### Study design and participants

The study was conducted in the Cajamarca region of Andean Peru, encompassing predominantly rural communities with high levels of mobile phone penetration. We implemented a three-arm, open-label cluster-randomized controlled trial across 164 clusters (communities). Eligible participants were mothers with children aged 3 to 9 months. Following baseline assessments, clusters were randomly assigned to the following: (i) a digital parenting chatbot intervention (DI), (ii) biweekly home visits (HVs), or (iii) a control group with no added services. The allocation ratio was 3.5:3.5:1, with a total of 2461 caregiver-child dyads (CCDs) enrolled: 1174 in DI, 317 in HVs, and 970 in control.

### AI-supported digital intervention

Caregivers in the DI group were introduced to a mobile-accessible parenting chatbot designed to deliver early learning guidance, activity suggestions, and health and parenting information tailored to their child’s age and developmental stage. The chatbot used an AI recommendation engine to select expert-generated content on the basis of caregiver engagement and reported feedback. Participants accessed the service through popular messenger platforms and received weekly push notifications suggesting developmentally appropriate activities. The chatbot also included a milestone tracker, question-and-answer features, and short articles on child development topics. Further details on the AI system are provided in section 1 in the Supplementary Materials.

### Home-visiting program

The HV group received in-person visits once every 2 weeks from trained community health workers following an adapted version of Peru’s national ECD curriculum, derived from the Reach Up model (*9*). Visits included structured activities, coaching on responsive caregiving, and the provision of toys and books. This model represents the current gold standard in parenting interventions but is operationally intensive and costly.

### Outcomes and measurement

The primary study outcome was children’s overall development at the age of 2.5 years using the Global Scales for Early Development (GSED). The GSED tool is a direct assessment tool evaluating children’s overall development (*10*). Secondary outcomes were caregiver stimulation, caregiver mental health, caregiver screen time, and caregiver reports of children’s development. Caregiver stimulation was measured through six questions regarding activities conducted with children in 3 days preceding the survey: reading or looking at picture books, telling stories, singing songs including lullabies, taking the child outside the home, playing with the child, and naming, counting, or drawing things with the child (*11*). We created a sum score for stimulation (ranging from 0 to 6) in a first step and then normalized this variable to zero mean and 1 SD. Caregiver mental health was measured using the DASS-21 (Depression Anxiety Stress Scales-21) scale (*12*, *13*). Caregiver-reported child development was assessed using the Caregiver-Reported Early Development Instruments (CREDI) short form (*14*). The caregiver screen time was self-reported by caregivers during the end-line assessment. Parenting beliefs, attitudes, and knowledge were measured through the Nurturing Parenting Beliefs and Behaviors Scale (NPBBS) (*15*). Parent-child interactions were measured through the Observation of Mother-Child Interaction (OMCI) tool (*16*).

### Sample size and analytical approach

The study was powered to detect a 0.25 SD difference in GSED *z*-scores between the DI group and the control group and a 0.50 SD *z*-score difference between the HV group and the control group. These calculations were based on an average of 15 children per study cluster, an intraclass correlation coefficient (ICC) of 0.12 found in a previous study (*17*), and an attrition rate of 15%. The HV minimum detectable effect size was based on two systematic reviews of home-visiting programs (*4*, *18*). We anticipated similar treatment effects among parents regularly interacting with the DI platform but anticipated only 50% compliance, yielding an anticipated intention-to-treat effect size of 0.25 SD.

Analyses followed an intention-to-treat framework using linear regression with cluster-robust standard errors. Covariates included child and caregiver demographics, baseline development scores, and household socioeconomic status (SES). Multiple imputation was used for missing covariate data. To assess spillovers, we asked all participants about familiarity with the DI platform at end-line. Last, to assess the relative cost effectiveness of the programs, we also calculated average intervention delivery cost, assuming program support for 18 months (ages of 6 to 24 months). Our primary cost-effectiveness measure was the average cost per SD improvement in child development from a provider perspective (*19*). We did not take caregiver time costs or other family benefits such as other children benefiting from interventions into consideration.

## RESULTS

A total of 4963 households were assessed for eligibility and 2461 households enrolled in the main study. Baseline interviews were conducted between 13 September 2021 and 3 March 2023. Nine hundred and seventy CCDs across 68 clusters were enrolled in the control arm (no eligible CCDs were found in four clusters allocated to this group), 1174 CCDs across 72 clusters in the DI arm, and 317 CCDs across 19 clusters in the HV arm (one cluster with zero enrollment). Four of 970 households in the control group erroneously received an intervention. In the DI arm, 166 of 1174 caregivers could not be linked to the platform because of absent phones or difficulties setting up the connection to the DI system. Caregivers were given a leaflet on how to link to the service later by themselves. Table S1 shows average characteristics of families enrolled compared to those not enrolled in the DI system: There were no differences in child age, sex, or birthweight, but families not enrolled had on average substantially lower education and SES and also had lower stimulation scores. Three of 317 households could not be reached for HVs. See fig. S1 for further details.

Table 1 describes baseline study characteristics. Fifty percent of children enrolled were female, and 7% were born with low birth weight. On average, mothers were 28 years old, and children were 5 months old at the baseline. Twenty-eight percent of mothers completed secondary education, and 24% received higher education. Ninety percent of children had a co-resident father. On average, children had one sibling and 2.5 adults in their household. Ninety-four percent of participating households could be contacted again at the end line.

**Table 1.** Baseline participant characteristics.

		Control		Home visits		Digital intervention		Overall	
	Clusters	68		19		72		159	
Child female	*N*, %	485	50.0%	151	47.6%	584	49.7%	1220	49.6%
Child birth weight <2.5 kg	*N*, %	71	7.4%	31	10.1%	85	7.4%	187	7.7%
Child age in month	Mean, SD	5.0	2.0	5.1	2.0	5.1	1.9	5.1	1.9
CREDI baseline *z*-score	Mean, SD	0.0	0.6	0.0	0.6	0.1	0.6	0.1	0.6
Mother age	Mean, SD	27.8	6.9	28.6	7.2	27.9	6.6	27.9	6.8
Mother basic education	*N*, %	475	49.8%	149	47.9%	538	46.7%	1162	48.1%
Mother sec. education	*N*, %	291	30.5%	77	24.8%	298	25.9%	666	27.6%
Mother higher education	*N*, %	187	19.6%	85	27.3%	316	27.4%	588	24.3%
Father present	*N*, %	865	89.4%	283	89.6%	1045	89.3%	2193	89.4%
Father age	Mean, SD	31.2	7.5	32.1	7.7	31.5	7.7	31.5	7.6
Father basic education	*N*, %	324	38.6%	101	37.8%	313	31.2%	738	35.0%
Father secondary education	*N*, %	336	40.0%	93	34.8%	396	39.4%	825	39.1%
Father higher education	*N*, %	179	21.3%	73	27.3%	295	29.4%	547	25.9%
Number of siblings	Mean, SD	1.1	1.1	1.2	1.3	1.1	1.1	1.1	1.2
Number of adults	Mean, SD	2.4	1.0	2.5	1.1	2.5	1.1	2.5	1.1
SES index*	Mean, SD	2.9	1.4	3.1	1.5	3.2	1.4	3.1	1.4
Home stimulation index	Mean, SD	3.4	1.3	3.4	1.2	3.5	1.2	3.5	1.2

* To quantify the household SES, we used principal components analysis of asset ownership variables available and divided households into five quintiles on the basis of the first principal component. *N* =2461.

End-line surveys were completed with 2183 households (88.5% of enrolled families) between 8 September 2023 and 17 March 2025. Ten children passed away, 100 households moved outside of the study area, and 65 households refused to participate in the end-line survey. The median (and mean) age of children at the end line was 29 months (min: 25; max: 41; interquartile range: 27 to 31). Table 2 shows the main trial results. Relative to the control group, HVs increased child development by 0.17 SD (95% CI: [0.03, 0.30]), while DI improved child development by 0.11 SD ([0.02, 0.19]) in fully adjusted models. Impacts on child development were robust to alternative standard error specifications (Table S2).

**Table 2.** Estimated impact on child development (GSED). This table shows intention-to-treat estimates of program impact on child development. The outcome variable is the *z*-score normalized within each age group in the sample. Estimates are based on ordinary least squares models with 95% confidence intervals on the basis of clustered standard errors. The estimated ICC was 0.005 ([0.0001, 0.117]). Three hundred and thirty-two observations were excluded from the analysis because of missing outcome data. Adjusted estimates in column 2 control for child age, child sex, low birth weight, baseline child developmental score (CREDI), mother age, mother education, the presence of father, father age, father education, baseline home stimulation score, household SES and household size.

	(1)	(2)
	Unadjusted	Adjusted
Home visit intervention	0.17**	0.17**
	(0.04–0.30)	(0.03–0.30)
Digital intervention	0.14***	0.11**
	(0.05–0.23)	(0.02–0.19)
Observations	2149	2149

** *P* < 0.05.

*** *P* < 0.01.

Effects of similar magnitudes were found for caregiver-reported child development, with HVs increasing child development by 0.10 SD ([−0.02, 0.22]) and DI increasing child development by 0.12 SD ([0.01, 0.23]) in the fully adjusted models (Table 3). No statistically significant associations were found between the intervention and caregiver mental health. We also found no associations with caregiver reported screen time in the adjusted model.

**Table 3.** Estimated impact on caregiver reported development, mental health and screen time. This table shows intention-to-treat estimates of program impact on secondary outcomes. Estimates are based on ordinary least squares models with clustered standard errors—numbers displayed correspond to point estimates and 95% confidence intervals. *P < 0.10; **P < 0.05; ****P < 0.01.

	(1)	(2)	(3)	(4)	(5)
	CREDI *z*-score	Caregiver depression	Caregiver anxiety	Caregiver stress	Caregiver screen time
Unadjusted estimates					
Home visit intervention	0.09	−0.03	−0.04	−0.00	−0.02
	(−0.03–0.21)	(−0.08–0.03)	(−0.09–0.02)	(−0.06–0.06)	(−0.23–0.19)
Digital intervention	0.15***	−0.01	−0.01	0.00	0.12*
	(0.05–0.25)	(−0.05–0.03)	(−0.05–0.03)	(−0.04–0.04)	(−0.00–0.25)
Adjusted estimates†					
Home visit intervention	0.10	−0.02	−0.03	−0.00	−0.07
	(−0.02–0.22)	(−0.08–0.04)	(−0.09–0.03)	(−0.06–0.05)	(−0.18–0.04)
Digital intervention	0.12**	0.00	−0.00	0.00	0.04
	(0.01–0.23)	(−0.04–0.04)	(−0.04–0.04)	(−0.04–0.04)	(−0.04–0.11)
Observations	2064	2069	2069	2069	2140

† Adjusted estimates in lower panel control for child age, child sex, low birth weight, baseline child developmental score (CREDI), mother age, mother education, the presence of father, father age, father education, baseline home stimulation score, household SES and household structure.

In terms of caregiver-child interactions, we found that HVs were associated with a 0.29 SD increase in caregiver-reported stimulation, while DI was associated with a 0.09 SD increase in caregiver-reported parenting beliefs in fully adjusted models. No statistically significant associations were found for caregivers’ observed emotional or cognitive support (Table 4).

**Table 4.** Estimated impact on caregiver-reported beliefs, behaviors, and observed interactions. This table shows intention-to-treat estimates of program impact on secondary outcomes. Estimates are based on ordinary least squares models with clustered standard errors—numbers displayed correspond to point estimates and 95% confidence intervals. *P < 0.05; **P < 0.05, ***P < 0.01.

	(1)	(2)	(3)	(4)
	Caregiver-reported stimulation	Caregiver-reported parenting beliefs (NPBBS)	Observed emotional support (OMCI)	Observed cognitive support (OMCI)
Unadjusted estimates				
Home visit intervention	0.33***	0.10	0.09	0.12
	(0.15–0.51)	(−0.14–0.35)	(−0.08–0.27)	(−0.03–0.27)
Digital intervention	0.13**	0.20***	0.06	0.09*
	(0.01–0.24)	(0.06–0.33)	(−0.04–0.17)	(−0.01–0.20)
Adjusted estimates†				
Home visit intervention	0.29***	0.05	0.07	0.11
	(0.20–0.39)	(−0.08–0.18)	(−0.06–0.19)	(−0.02–0.23)
Digital intervention	0.04	0.09*	0.02	0.07
	(−0.05–0.12)	(−0.00–0.18)	(−0.08–0.11)	(−0.04–0.17)
Observations	2126	2123	2157	2157

† Adjusted estimates in lower panel control for child age, child sex, low birth weight, baseline child developmental score (CREDI), mother age, mother education, the presence of father, father age, father education, baseline home stimulation score, household SES and household structure.

Table S3 shows per-protocol analysis results. Estimated treatment effects were very similar to the intention-to-treat estimates displayed in Tables 2 to 4. We found little evidence of spillovers for DI: At the end-line, 96% of caregivers in the control and 98% of caregivers in the HV arm reported to never have heard of the platform.

**Fig. 1.**
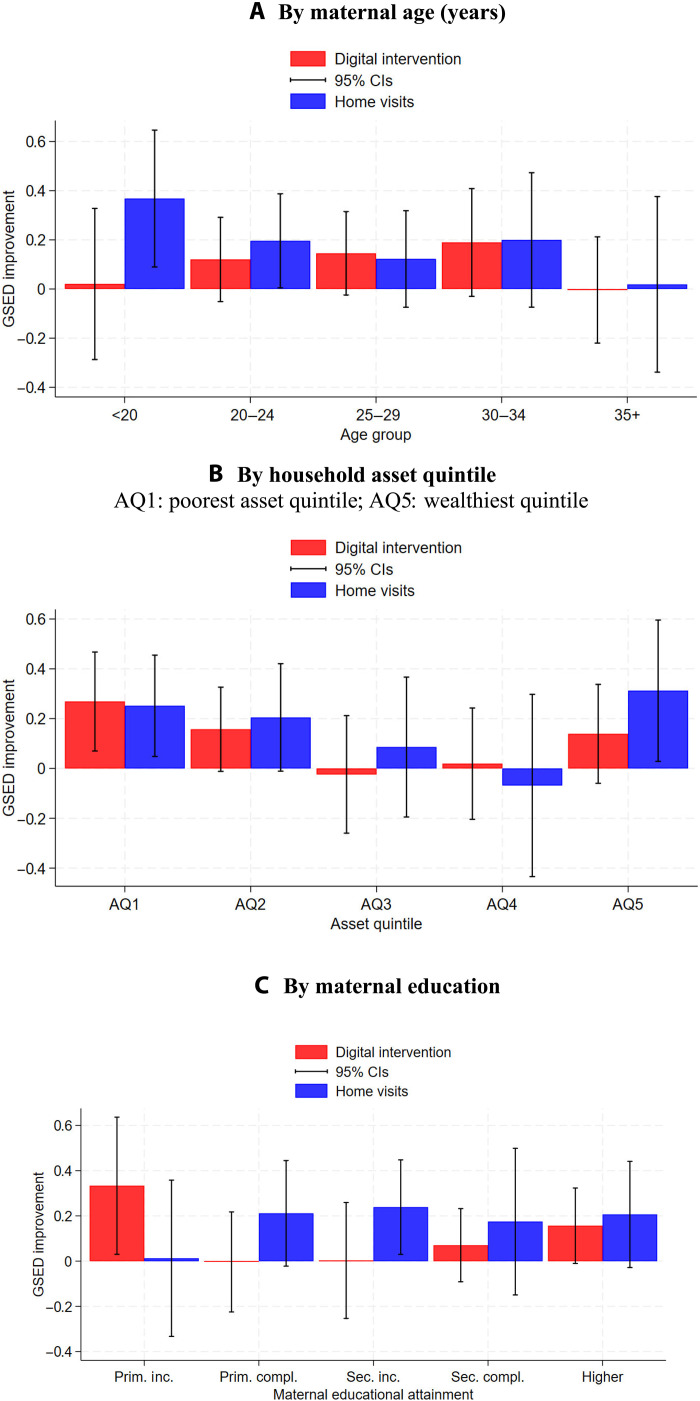
Stratified program impact on the GSED *z*-score. (**A** to **C**) This figure shows estimated subgroup impacts using fully adjusted regression models with clustered standard errors.

Figure 1 shows estimated subgroup impacts for both interventions. Estimated confidence intervals were large and overlapping across subgroups. In terms of age, DI appears to have had the largest impact among mothers in the 20-to-34 age range, while HVs showed positive changes for all but the oldest (35+) age group. For wealth (asset quintile) and education, DI appears to have had the largest impacts at the bottom and top ends of the distribution and small impacts in the middle. HVs displayed a similar pattern for wealth but slightly more constant impacts across education groups, with much smaller impacts for the least educated. Tables S4 to S6 show full results underlying Fig. 1.

Table 5 shows estimated costs per child for the two experimental interventions. For HVs, the primary cost factors were staff time, transport, and intervention materials; for DI, costs include an initial setup visit, a small booklet with reenrollment instructions, costs for sending push messages, and other supporting materials. With an average cost of USD 654 per child over 18 months, the HV intervention cost was similar to Peru’s national home-visiting program cost estimates previously published (*20*) and roughly 15 times the cost of DI (USD 41.4). Table S7 provides further details on DI costs per child for a larger national program covering all children in Peru. While initial setup costs do not vary much, all IT (information technology)-related costs go down substantially with a larger program. When benchmarked against developmental gains for cost-effectiveness analysis, the digital intervention achieved an estimated cost of USD 414 per 1 SD increase in development compared to USD 4090 for HVs.

**Table 5.** Estimated program cost per child. This table shows estimated program costs per child in USD. Calculations were based on 18 months of interventions. For HVs, costs were based on two field workers jointly supporting 80 families residing in rural areas using motorcycles for transport. For both programs, costs shown are based on a medium scale program reach of ~25,000 children.

	Home visits	Digital intervention
Staff salary incl. social contributions	290.5	19.7*
Transport	51.8	0.0
Equipment	1.7	4.7
Toys and materials	135.1	0.0
IT infrastructure	0	7.9
Push notifications	0	7.0
Content development and support	0	2.2
Staff management cost	175.28	0.0
Total cost in USD	654.4	41.4
Estimated cost per SD increase in child development in USD	4090	414
95% confidence intervals	[2181, 136,340]	[218, 2072]

* Estimated cost of initial HV and booklet.

## DISCUSSION

This study provides large-scale, population-level evidence that an AI-enhanced chatbot can positively affect child development in a resource-constrained setting*.* Although traditional HVs produced somewhat larger developmental gains, digital intervention achieved improvements in child development at much lower cost. This represents a significant advancement in the search for scalable ECD solutions.

The findings affirm the feasibility of digital delivery even in rural, low-income contexts, where more than 90% of families owned compatible mobile phones. Engagement with the chatbot was high, and intervention proved relatively accessible across diverse subgroups. We found that ~15% of families with on average lower SES were not able to enroll into the DI system, suggesting that complementary programs targeting some of the most vulnerable population could be beneficial to avoid unintentionally widening inequities. Despite this, benefits for DI were visible across the entire SES spectrum.

From a policy perspective, AI-enabled parenting support offers an appealing complement to existing service models. Its lower marginal cost and minimal infrastructure requirements make it especially attractive for regions facing human resource constraints or aiming to reach dispersed populations.

### Limitations

Several limitations warrant consideration. First, the study evaluated only one specific digital platform in one specific (high-altitude Andean) setting; results may not generalize to other designs (e.g., designs not providing onboarding supports or booklets for reenrollments), chatbot architectures, or contexts. Second, while phone ownership was high, we cannot exclude digital access barriers in more remote or less connected regions. Third, our design does not identify the minimal effective package—future work should test which components (e.g., onboarding, push notifications, and chatbot question and answer) are necessary to drive impact. Fourth, our cost-effectiveness analysis focused exclusively on the provider perspective and did not take caregiver time and efforts into consideration. Given that all families already had a phone with a data plan, we did not consider these costs that might occur in other settings. We also did not consider other benefits of HVs such as potential emotional support for caregivers, other children in the household, or neighborhoods benefitting from the toys delivered as part of HV intervention.

Moreover, our sample was not powered to detect differential impacts across all subgroups. Additional research is needed to explore the heterogeneity of treatment effects and the role of caregiver characteristics in mediating outcomes.

Overall, the results from this trial suggest that digital parenting chatbots, powered by AI and delivered through widely used mobile platforms, can effectively enhance early child development in low-resource settings. While they may not be able to fully replace in-person support, such tools offer a cost-effective, scalable strategy to augment parenting interventions globally. As AI technologies continue to evolve, their thoughtful integration into the ECD ecosystem could transform the landscape of early life support.

## MATERIALS AND METHODS

### Public involvement

As described in further detail by Jäggi *et al.* (*21*), local families were involved in the project from the beginning. All content used on the DI platform was carefully reviewed by our study team for accuracy and contextual relevance. To ensure local relevance, we also reviewed and discussed selected content and platform features in focus groups and interviews with local mothers. The study was also introduced to national and local authorities as well as the national ECD program (Cuna Más) at the beginning of the trial, and periodic updates were provided throughout the study period.

### Trial design

The study was designed as an open-label, parallel cluster-randomized controlled trial. Given the much higher cost of the home visiting arm, clusters were randomly assigned to control, DI, and HVs at a ratio of 3.5:3.5:1.

### Changes to trial protocol

No changes were made to the trial protocol.

### Trial setting

This trial was conducted in three provinces of the Cajamarca region in northern Andean Peru located between 1900 and 3900 m above sea level: Cajabamba, Cajamarca, and San Marcos. The study area is predominantly rural with small settlements scattered within a 1.5-hour travel time around the cities of Cajabamba, Cajamarca, and San Marcos. The region is representative of many rural and peri-urban settings in Andean South America, with most households located in remote areas and relying on agriculture.

### Intervention and comparator

The trial was designed to assess the (absolute and relative) effectiveness of two interventions: in person HVs through trained child development facilitators and digital support provided through an AI-supported chatbot. HVs were conducted by trained study staff every 2 weeks for 18 months following an adapted version of the national Cuna Más curriculum (*22*). This curriculum is based on the original Reach Up materials (*9*, *23*) developed in Jamaica, which defines activities for each visit and also provides participating families with books and age-specific toys to support these activities. Digital intervention (DI) was provided through the Afini platform, an AI-supported chatbot for early childhood development providing personalized caregiver guidance through automated conversational interaction and content recommendation. At the beginning of the study, selected families were visited by study staff and introduced to the platform. Caregivers were directly connected to the chatbot by study staff, who registered caregivers in the system using a unique study ID. Once registered, caregivers could interact with the platform through a messenger service (Facebook or Whatsapp) and/or a separately installed mobile app. To support families without reliable digital access or in cases of lost or defective phones, all families received a printed booklet. This booklet included activity guides and flash cards with step-by-step guidance on how to reconnect to the digital platform if needed.

The system was deployed across three delivery channels: WhatsApp, Facebook Messenger, and a mobile application; and supported by a backend microservices architecture developed in Python using the Django framework. The following versions were used during the randomized trial: iOS app version 1.2.0 (released 3 April 2023) and iOS app version 1.2.2 (released 14 April 2023). Although no formal version locking was implemented, the intervention features, content library, and core functionalities remained stable throughout the study period. Please see the Supplementary Materials for additional information. 

In addition, all caregivers in the DI condition received weekly push notifications with brief messages designed to encourage engagement with the platform and get age-appropriate activities recommended for their children. Recommended activities were generated by human experts and chosen by the AI system on the basis of children’s age and development as well as on the basis of caregivers’ prior feedback on proposed activities. Through the platform, caregivers also had access to a developmental milestone tracker and short articles discussing common child development challenges, which they could access at their convenience.

The comparator for both interventions was a control group not receiving any additional support. Families in the control group were visited by the study team only to collect data and assess child development at the beginning (baseline) and end (end-line) of the study.

### Outcomes

The primary study outcome was children’s overall development at the age of 2.5 years using the GSED. The GSED tool is a direct assessment tool evaluating children’s overall development (*10*). Given that developmental scores increase with age, we used age-normalized *z*-scores as our primary outcome measure. Secondary outcomes were caregiver stimulation, caregiver mental health, caregiver screen time, and caregiver reports of children’s development. Caregiver stimulation was measured through six questions regarding activities conducted with children in 3 days preceding the survey: reading or looking at picture books, telling stories, singing songs including lullabies, taking the child outside the home, playing with the child, and naming, counting, or drawing things with the child (*11*). We created a sum score for stimulation (ranging from 0 to 6) in a first step and then normalized this variable to a zero mean and 1 SD. Caregiver mental health was measured using the DASS-21 scale (*12*, *13*). Caregiver-reported child development was assessed using the CREDI short form (*14*). The caregiver screen time was self-reported by caregivers during the end-line assessment. Parenting beliefs, attitudes, and knowledge were measured through the NPBBS (*15*). Parent-child interactions were measured through a locally adapted version of the OMCI tool (*16*).

### Harms

Given the noninvasive nature of both intervention arms, no harm was anticipated in this trial. No unexpected trial-related events were reported during the study period.

### Sample size

The study was powered to detect a 0.25 SD difference in GSED *z*-scores between the DI group and the control group and a 0.50 SD *z*-score difference between the HV group and the control group. These calculations were based on an average of 15 children per study cluster, an ICC of 0.12 found in a previous study (*17*), and an attrition rate of 15%. The HV minimum detectable effect size was based on two systematic reviews of home-visiting programs (*4*, *18*). We anticipated similar treatment effects among parents regularly interacting with the DI platform but anticipated only 50% compliance, yielding an anticipated intention-to-treat effect size of 0.25 SD.

### Randomization

Randomization was done at the cluster level using a random number draw generated in the Stata SE 18.0 software package by the first author (*24*). Each cluster corresponded to a village in rural areas and to blocks in urban areas. Given that the implementation cost of HVs was substantially higher than the cost of the two other arms, and given that we expected HVs to have twice the effect size of DI, we decided to allocate 70 clusters each to control and DI and 20 clusters only to HVs. Randomization was stratified at the province level, enforcing a 3.5:3.5:1 ratio in each of the three provinces (Cajabamba, Cajamarca, and San Marcos).

### Allocation concealment mechanism and blinding

Given the nature of the two interventions, blinding of participating families was not possible. To minimize analyst bias, we used a blinded dataset for the initial analysis and only unblinded the dataset once the final models and analyses were completed. Field workers conducting the child assessments were not informed about the random allocation at the end-line; full blinding of field workers was not feasible in practice given that caregivers may have commented on specific interventions they received and informal exchanges among field workers may also have happened.

### Ethics and human subjects

The study was approved by the Ethics Committee for North-Western Switzerland as well as the Comité Institucional de Ética en Investigación of Cayetano Heredia University (202522). All participating caregivers provided written informed consent to participate in the study.

### Recruitment

Caregivers were recruited through door-to-door visits by study staff. We obtained a list of families with children under the age of 1 year from the district health office and visited families to verify eligibility. Families were eligible if they were not planning to move outside of the study area in the coming year and not receiving other parenting interventions. Eligible families were informed about the study and enrolled upon their consent to participate.

### Study timeline

Study enrollment and baseline interviews were conducted between 13 September 2021 and 3 March 2023. Treatments were initiated immediately upon study enrollment in the two intervention arms. End-line assessments were conducted between 8 September 2023 and 17 March 2025.

### Statistical methods

Intention-to-treat models were used to estimate the impact of both interventions. Given the continuous nature of the primary outcome variable, linear regression models were used to assess primary treatment effects. To account for the cluster-level treatment assignment, standard errors were computed using Huber’s cluster-robust sandwich estimator (*25*, *26*). We also estimated intention-to-treat models with an extensive list of baseline covariates, including child age, child sex, low birth weight (child birth weight <2.5 kg), baseline developmental score (CREDI), mother age, mother education, the presence of father, father age, father education, baseline home stimulation score (*11*), household SES, and the number of children and adults in the household.

Missing covariate (but not outcome) data were imputed using multiple imputations by chained equations. For all estimates presented here, 250 imputations were generated. No multiple hypothesis correction was applied to secondary outcomes, which we present as exploratory associations.

To assess the relative impact of both arms across subpopulations, exploratory, and not prespecified, subgroup models were estimated, stratifying the sample by caregiver age, educational attainment, and household SES. To classify household’s SES, we conducted principal components analysis of assets owned and then divided households into five quintiles on the basis of the first principal component (*27*).

We also estimated per-protocol models to assess the relative impact among compliers: For both groups, we defined compliance as participating in the intervention for at least 6 months. For HVs, this corresponds to 13 completed visits. For DI, compliance was defined as users engaging with the chatbot (responding to push-messages, opening activities, or requesting information) over a period of at least 6 months.

Last, to assess the relative cost effectiveness of the programs, we also calculated average intervention delivery cost, assuming program support for 18 months (ages of 6 to 24 months). Our primary cost-effectiveness measure was the average cost per SD improvement in child development (*19*). We base these estimates on our main adjusted impact estimates reported as well as the corresponding standard errors. We do not take uncertainty related to cost estimates into consideration.

## Supplementary Materials

### This PDF file includes:

AI description Figs. S1 to S3 Tables S1 to S7
