# Supplementary material for: Digital support systems to improve child development in Peru: A cluster-randomized controlled open-label trial
Source: Sci Adv. 2026 Mar 4;12(10):eaeb9403. doi: 10.1126/sciadv.aeb9403 (PMC12959410; doi:10.1126/sciadv.aeb9403)
Supplement: Supplementary file 1 — AI description Figs. S1 to S3 Tables S1 to S7 [file sciadv.aeb9403_sm.pdf]

Science Advances
AAAS

Supplementary Materials for

Digital support systems to improve child development in Peru: A cluster-randomized controlled open-label trial

Günther Fink *et al.*

Corresponding author: Günther Fink, guenther.fink@swisstph.ch

Sci. Adv. **12**, eaeb9403 (2026)
DOI: 10.1126/sciadv.aeb9403

This PDF file includes:

AI description
Figs. S1 to S3
Tables S1 to S7

Supplementary Materials: AI description

Model Description

The DI model exclusively used to recommend pre-created educational content from a human expert-curated library of over 1,500 activities and messages. Each piece of content has been developed and validated by experts in early childhood development.
It consists of two layers: the Natural Language Understanding (NLU) layer, which serves as the interface to the user and an AI-driven content recommendation engine, which ranks and selects expert-curated content for the users.
The Natural Language Understanding (NLU) layer in Afini Connect is not proprietary; it uses RASA, an open-source model, which operates through a session-tree logic designed to guide user interactions in a structured and predictable way. The proprietary component of the system lies in the AI-driven content recommendation engine, which determines the most relevant materials to deliver to each family.

Core Model Characteristics

Model Type: Proprietary recommendation system that combines demographic, sequential behavioral, and contextual data to select content most relevant to each child's age, developmental level, and engagement history. The system uses a collaborative filtering model, and a behavior sequence transformer model.

AI-driven content recommendation engine usage: The AI recommendation model is used exclusively for recommendation ranking, not for generating or modifying content.

Content Source: All content is internally developed by human experts and pre-approved before being entered into the system. The recommendation model only selects content from this fixed library.

Technical and Operational Details

Model Family and Provenance: The underlying recommendation algorithm is proprietary to Afini Connect. No generative AI model or external language model is used in user interactions.

Training Data: Model optimization relies solely on anonymized demographic data (e.g., age, gender), contextual data (e.g. activities metadata), and engagement data (e.g., click-through rates, completion patterns) from Afini's own platform. No external or personally identifiable data is used for training.

Version Locking During Trials: No formal version locking mechanism was implemented during the trial. However, the study operated using stable production versions of all the channels, with minor iterative updates (e.g., bug fixes and performance improvements) that did not modify the intervention logic, content structure, or underlying AI recommendation system.

Update Cadence: Algorithm parameters are revised based on aggregated usage analytics. The content library is reviewed and updated monthly.

Latency and Uptime: The system maintains a latency of under 1 second for recommendations and achieves 98.88% uptime through its cloud-based infrastructure.

Multilingual Handling: Afini Connect supports Spanish, Portuguese, and English, automatically adapting recommendations and content versions to the user's region and language preference.

Guardrails and Human Oversight

Recommendations are strictly limited to pre-validated age-specific content (No generative content, i.e. through Large Language Models (LLMs) was used).

The system cannot produce or modify text responses beyond recommending content from the pre-approved library.

Confidence thresholds trigger fallback mechanisms (e.g., generic age-based suggestions).

Failure Modes:

If the recommender system cannot produce a confident match due to missing or inconsistent data, it defaults to safe, developmentally appropriate content (i.e., generic age-based suggestions).

Human-in-the-Loop Escalation:

Every chat includes a visible "Escalate to Human" button. When activated, the user is connected with a trained educator or coach for direct human support. In particular questions some of the branches in Rasa would defer/recommend the user to visit the local healthcare facility or allow a human in the "support center" to take over the chat

Supplemental Materials Figure S1: Consort Flow Chart

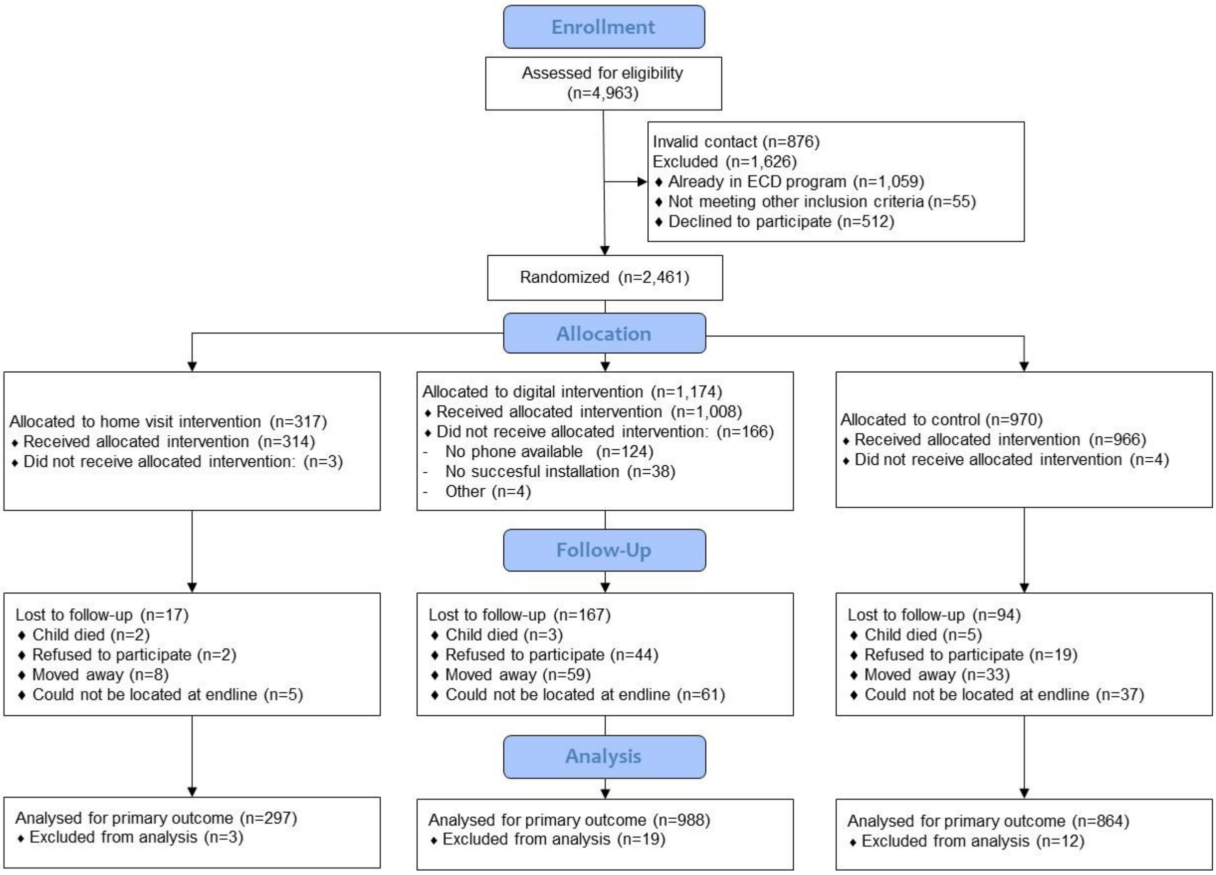

Supplemental Materials Figure S2: Follow-up rates by group

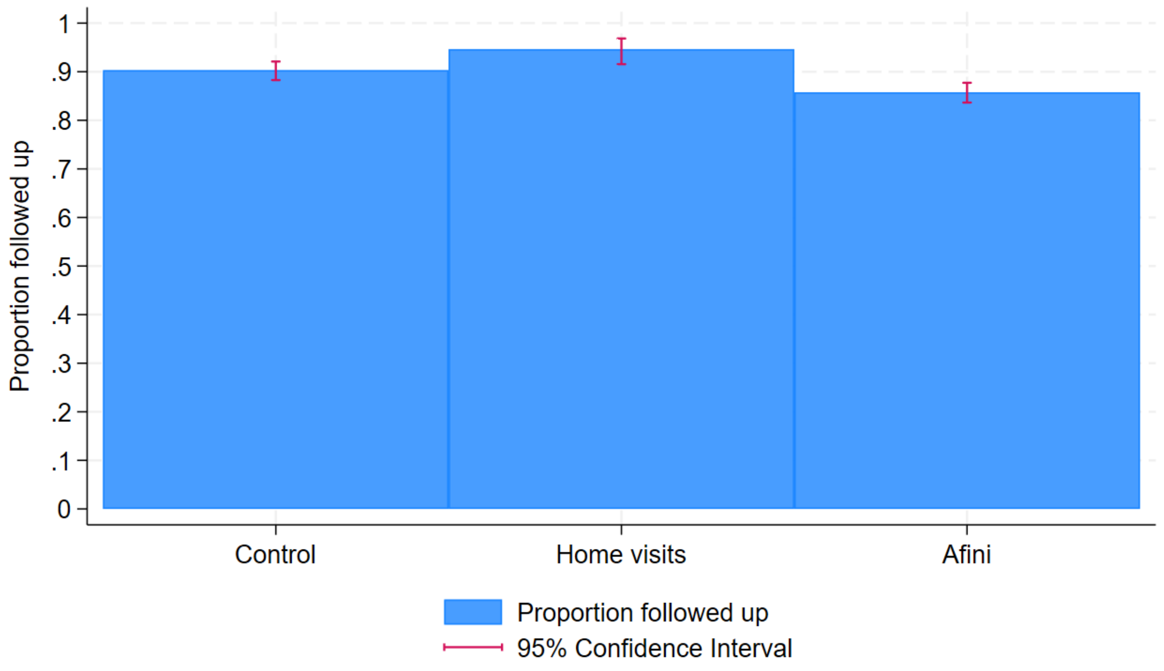

Notes: Figure S2 show the proportion of caregiver-child dyads followed up by arm.

Supplemental Materials Figure S3: Weeks active in the DI Arm

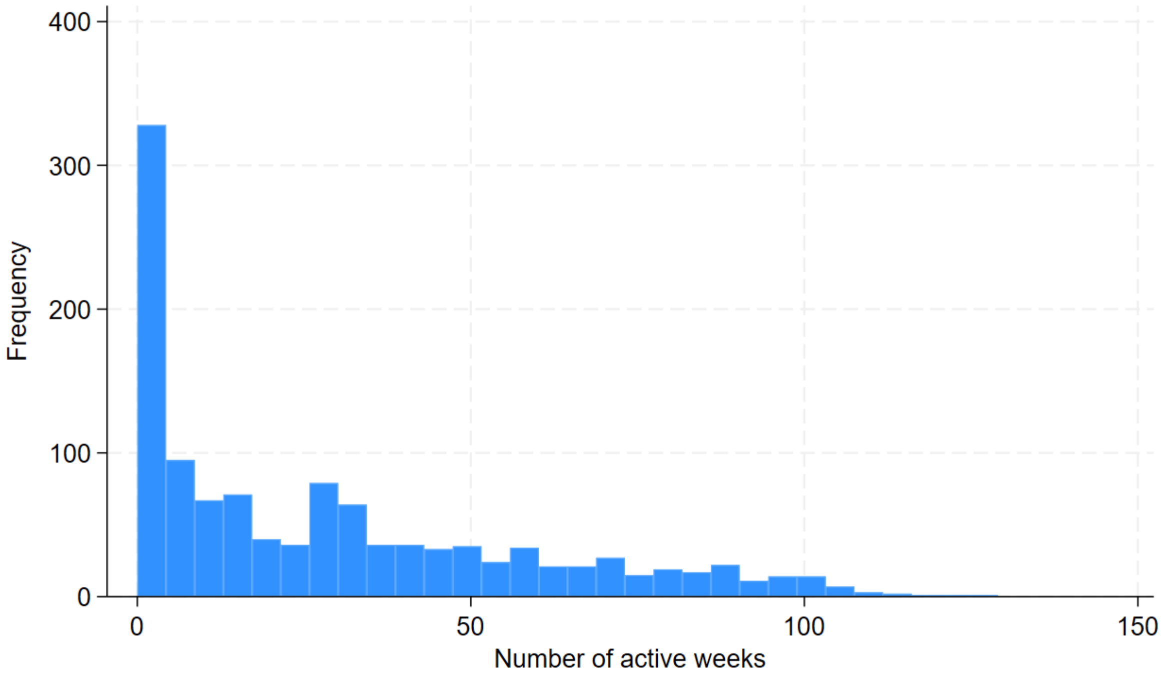

Notes: Figure S3 show the number of weeks users in the DI group were active.

Supplemental Materials Table S1: Comparison of families successfully enrolled and not enrolled into DI

	Stats	Enrolled		Not enrolled		H0: same mean p-value
Child female	N, %	499	49.5%	85	51.2%	0.67
Child birth weight < 2.5kg	N, %	72	7.3%	13	8.0%	0.75
Child age in month	Mean, SD	5.1	0.0	5.0	1.2	0.88
Child infant development	Mean, SD	0.1	0.0	0.0	0.0	0.04
Mother age	Mean, SD	27.9	1.9	28.2	1.9	0.61
Mother incompl. sec. or less	N, %	422	42.5%	116	73.4%	0.00
Mother secondary completed	N, %	272	27.4%	26	16.5%	0.00
Mother higher education	N, %	300	30.2%	16	10.1%	0.00
Father present	N, %	894	89.0%	151	91.5%	0.28
Father age	Mean, SD	31.5	0.6	31.9	0.6	0.59
Father incompl. sec. or less	N, %	243	28.3%	70	48.3%	0.00
Father secondary completed	N, %	343	39.9%	53	36.6%	0.42
Father higher education	N, %	273	31.8%	22	15.2%	0.00
Number of siblings	Mean, SD	1.0	6.5	1.3	7.0	0.00
Number of adults	Mean, SD	2.5	7.6	2.3	8.4	0.01
SES index	Mean, SD	3.3	1.1	2.5	1.2	0.00
Home stimulation index	Mean, SD	3.6	1.1	3.2	0.9	0.01
Cajabamba province	N, %	307	30.5%	49	36.7%	0.82
San Marcos province	N, %	131	13.0%	40	15.0%	0.05

Table S1 compares mean characteristics of families successfully enrolled into the DI system to those of families not enrolled.

Supplemental Materials Table S2: Robustness checks

	Outcome: GSED z-score		
	(1)	(2)	(3)
Model	Wild bootstrap	Random effects	Cluster averages
Home visits intervention	0.167**	0.167**	0.212**
p-value:	0.015	0.013	0.012
Digital intervention	0.139***	0.139***	0.170**
p-value:	0.003	0.002	0.002
Observations	2,151	2,151	159

Table S2 shows intention-to-treat estimates of program impact on child development. The outcome variable is z-score normalized within each age group in the sample. Estimates are based on ordinary least squares models and not adjusted for covariates. Column 1 shows primary estimates with cluster wild bootstrapped standard errors; column 2 shows primary estimates with mixed models (cluster random effects) and column 3 shows an analysis of cluster-level average outcomes.

Supplemental Materials Table S3: Per protocol analysis

	(1)	(2)	(3)	(4)	(5)	(6)
Outcome	GSED	CREDI	Caregiver depression	Stimulation (MICS)	Parenting beliefs, attitudes, knowledge (NPBBS)	Screen time
Home visits	0.13*	0.14***	-0.05	0.34***	0.10	-0.12**
	(-0.00 - 0.26)	(0.04 - 0.24)	(-0.11 - 0.01)	(0.22 - 0.46)	(-0.05 - 0.24)	(-0.21 - -0.03)
Digital intervention	0.09*	0.10**	-0.00	0.05	0.11**	0.07
	(-0.00 - 0.19)	(0.01 - 0.18)	(-0.04 - 0.04)	(-0.04 - 0.14)	(0.02 - 0.21)	(-0.02 - 0.16)
Observations	1,787	1,730	1,738	1,777	1,775	1,779

Notes: Table S3 shows the results of the per protocol analysis. Estimates are based on ordinary least squares models with clustered standard errors – numbers displayed correspond to point estimates and 95% confidence intervals. All estimates are adjusted for child age, child sex, low birth weight, baseline developmental score (CREDI), mother age, mother education, presence of father, father age, father education, baseline home stimulation score, household SES and household structure. For the DI arm, participants were considered active on average for 29 weeks; 69% of subjects were considered compliant (active for six months). SF3 shows the distribution of weeks of activity. For the HV arm, the average number of sessions was completed was 17; 66% of subjects were considered compliant.

Supplemental Materials Table S4: Age-group specific impacts on child development (in months)

Maternal Age Stratum	Ages 15-19	Ages 20-24	Ages 25-29	Ages 30-34	Ages 35+
Home visits intervention	0.25**	0.20*	0.09	-0.07	0.31**
	(0.05 - 0.45)	(-0.01 - 0.42)	(-0.19 - 0.37)	(-0.43 - 0.30)	(0.03 - 0.60)
Digital intervention	0.27***	0.16*	-0.02	0.02	0.14
	(0.07 - 0.47)	(-0.01 - 0.33)	(-0.26 - 0.21)	(-0.20 - 0.24)	(-0.06 - 0.34)
Observations	406	408	432	446	448

Notes: Table S4 shows intention-to-treat estimates by maternal age group (in years). Outcomes are GSED z-scores. Estimates are based on ordinary least squares models with clustered standard errors – numbers displayed correspond to point estimates and 95% confidence intervals. All estimates are adjusted for child age, child sex, low birth weight, baseline developmental score (CREDI), mother age, mother education, presence of father, father age, father education, baseline home stimulation score, household SES and household structure.

Supplemental Materials Table S5: SES group specific impacts on child development

Stratum	Quintile 1 (lowest)	Quintile 2	Quintile 3	Quintile 4	Quintile 5 (highest)
Home visits intervention	0.17*	0.22**	0.05	-0.08	0.33**
	(-0.01 - 0.35)	(0.00 - 0.44)	(-0.20 - 0.30)	(-0.42 - 0.26)	(0.02 - 0.64)
Digital intervention	0.23**	0.16*	-0.00	0.06	0.14
	(0.04 - 0.42)	(-0.00 - 0.32)	(-0.23 - 0.22)	(-0.13 - 0.26)	(-0.05 - 0.34)
Observations	406	408	434	446	449

Notes: Table S5 shows intention-to-treat estimates by asset quintile. Outcomes are GSED z-scores. Estimates are based on ordinary least squares models with clustered standard errors – numbers displayed correspond to point estimates and 95% confidence intervals. All estimates are adjusted for child age, child sex, low birth weight, baseline developmental score (CREDI), mother age, mother education, presence of father, father age, father education, baseline home stimulation score, household SES and household structure.

Supplemental Materials Table S6: Educational attainment group specific impacts on child development

Stratum	Incomplete primary or less	Primary completed	Incomplete secondary	Complete secondary	Higher
Home visits intervention	0.01	0.21*	0.24**	0.17	0.21*
	(-0.34 - 0.36)	(-0.02 - 0.45)	(0.03 - 0.45)	(-0.15 - 0.50)	(-0.03 - 0.44)
Digital intervention	0.33**	-0.00	0.00	0.07	0.16*
	(0.03 - 0.64)	(-0.23 - 0.22)	(-0.26 - 0.26)	(-0.09 - 0.23)	(-0.01 - 0.32)
Observations	303	409	287	587	528

Notes: Table S6 shows intention-to-treat estimates by educational attainment group. Outcomes are GSED z-scores. Estimates are based on ordinary least squares models with clustered standard errors – numbers displayed correspond to point estimates and 95% confidence intervals. All estimates are adjusted for child age, child sex, low birth weight, baseline developmental score (CREDI), mother age, mother education, presence of father, father age, father education, baseline home stimulation score, household SES and household structure.

Supplemental Materials Table S7: Estimated program cost per child at 550,000 children in Peru

Item	Cost	Description
Enrollment cost (one hour in person visit + print booklet)	**$16.68**	$12.62/hr per visit plus $4 per user booklet
Push notifications fees	**$7.00**	70–78 pushes per user over 18 months at $0.08–$0.10 each
DI family platform and content	**$0.20**	$6,000/month spread across 550,000 users for 18 months
Server hosting and data storage	**$0.22**	$3,000/month allocated per every 25,000 users ($66,000 per month) over 18 months
Monitoring platform	**$0.02**	$600/month across 550,000 users for 18 months.
Content updates and maintenance	**$1.96**	30,000- 60,000 USD per month among 50k users times 18 months
Initial set-up	**$0.09**	$165,000 one-time fee depreciated over 5 years, apportioned for 18 months
Total	**$26.16**	
